# Supplementary material for: Generation of Fibrotic Liver Organoids Using Hepatocytes, Primary Liver Sinusoidal Endothelial Cells, Hepatic Stellate Cells, and Macrophages
Source: Cells. 2023 Oct 24;12(21):2514. doi: 10.3390/cells12212514 (PMC10647544; doi:10.3390/cells12212514)
Supplement: Supplementary file 1 [file cells-12-02514-s001.zip › cells-2625656-supplementary.pdf]

Supplementary Table S1. Primer sequences used in this study.

Gene name	Sequence (5′-3′)		Accession #
GAPDH	Forward	CAAGGCTGAGAACGGGAAGC	NM_001256799.3
	Reverse	AGGGGGCAGAGATGATGACC	
AFP	Forward	AGTGAGGACAAACTATTGGCCT	NM_001354717.2
	Reverse	ACACCAGGGTTTACTGGAGTC	
ALB	Forward	TTTATGCCCCGGAACTCCTTT	NM_000477.7
	Reverse	AGTCTCTGTTTGGCAGACGAA	
CYP3A4	Forward	AAGTCGCCTCGAAGATACACA	NM_001202855.3
	Reverse	AAGGAGAGAACACTGCTCGTG	
CYP3A7	Forward	TGCTTTGTCCTTCCGTAAGGG	NM_000765.5
	Reverse	CAGCATAGGCTGTTGACAGTC	
CYP2C9	Forward	GCCTGAAACCCATAGTGGTG	NM_000771.4
	Reverse	GGGGCTGCTCAAAATCTTGATG	
CYP2D6	Forward	TGGCAAGGTCCTACGCTTC	NM_000106.6
	Reverse	GCCACCACTATGCACAGGTT	
G6PC1	Forward	CTACTACAGCAACACTTCCGTG	NM_000151.4
	Reverse	GGTCGGCTTTATCTTTCCCTGA	
GLUT2	Forward	GGGCAATTATGATCTGTGGCA	NM_000340.2
	Reverse	TTCTGCTCACTCGATGCTTCT	
HNF4a	Forward	CACGGGCAAACACTACGGT	NM_178849.3
	Reverse	TTGACCTTCGAGTGCTGATCC	
UGT2B7	Forward	GATCCCAACAACTCATCCGCT	NM_001074.4
	Reverse	CAGCAGCTCACTACAGGGAA	
KRT19	Forward	GTCACAGCTGAGCATGAAAGC	NM_002276.5
	Reverse	GCTCACTATCAGCTCGCACA	
SOX9	Forward	GGAAGTCGGTGAAGAACGGG	NM_000346.3
	Reverse	TGTTGGAGATGACGTCGCTG	
AXIN2	Forward	AGCTTACATGAGTAATGGGG	NM_004655.4
	Reverse	AATTCCATCTACACTGCTGTC	
iNOS	Forward	GTGCAAACCTTCAAGGCAGC	NM_000625.4
	Reverse	CTTGGCCATCCTCACAGGAG	
IL-1β	Forward	CAGGCTGCTCTGGGATTCTC	NM_000576.3

	Reverse	GTCCTGGAAGGAGCACTTCAT	
TNF-α	Forward	CCCATGTTGTAGCAAACCCT	NM_000594.4
	Reverse	TGAGGTACAGGCCCTCTGAT	
IL-6	Forward	GTAGCCGCCCCACACAGACAGCC	NM_000600.5
	Reverse	GCCATCTTTGGAAGGTTC	
IL-10	Forward	TTCGAGATCTCCGAGATGCC	NM_000572.3
	Reverse	AGTTCACATGCGCCTTGATG	
IL-4	Forward	AGGAAACCTTCTGCAGGGCT	NM_000589.3
	Reverse	CTCTCTCATGATCGTCTTTAGCCT	
TGF-β	Forward	AAGTGGACATCAACGGGTTC	NM_000660.7
	Reverse	GTCCAGGCTCCAAATGTAGG	
CD206	Forward	GTGATGGGACCCCTGTAACG	NM_002438.4
	Reverse	CTGCCCAGTACCCATCCTTG	
CD31	Forward	AACGGAAGGCTCCCTTGATG	NM_000442.5
	Reverse	TAAGAACCGGCAGCTTAGCC	
LYVE1	Forward	GAGCAAAAAGGCGAACCAGC	NM_006691
	Reverse	CAACCCAGCCATAGCTGCAAG	
VEGF-A	Forward	ACATCACCATGCAGATTATGCG	NM_003376.6
	Reverse	ACCGGGATTTCTTGCGCTTT	
VEGFR2	Forward	ACCGGCTGAAGCTAGGTAAG	NM_002253.4
	Reverse	CGATGCTCACTGTGTGTTGC	
eNOS	Forward	GCCGGAACAGCACAAGAGTTAT	NM_000603.5
	Reverse	AGCCCGAACACACAGAACC	
EDNRB	Forward	CAATGTCTACAAGCTGCTGGC	NM_003991.4
	Reverse	GCTGTCCATTTTGGAACCCC	
α-SMA	Forward	GACAATGGCTCTGGGCTCTGTAA	NM_001613.4
	Reverse	CTGTGCTTCGTCACCCACGTA	
COL1A1	Forward	CAGGAGGCACGCGGAGTGTG	NM_000088.3
	Reverse	GGCAGGGCTCGGGTTTCCAC	
COL2A1	Forward	TTCCCCCTCCTGCTCCAAG	NM_001844.5
	Reverse	GCCAGCCTCCTGGACATC	
COL3A1	Forward	TCCCGGTCCTGCTGGTTCCC	NM_000090.4
	Reverse	ATGGCAGCGGCTCCAACACC	
COL4A1	Forward	TTTCGACTTGCGGCTCAAAG	NM_001845.6

	Reverse	CAATCCTACAGAACCCGGCG	
COL4A2	Forward	GAGGCTAAGTGGGACTGACC	NM_001846.4
	Reverse	AAACTTCTTCACACCCGCCA	
COL5A3	Forward	AGTTTCCCGCGGACCC	NM_015719.4
	Reverse	ACATCCACAGGATCGGCCT	
COL6A1	Forward	CACTCAAAAGCAGCGTGGAC	NM_001848.3
	Reverse	GTCGGTCACCACAATCAGGT	
COL6A2	Forward	AACATGACGCTGTTCTCCGA	NM_001849.4
	Reverse	CACGGACAGCTCTGTTTGG	
COL6A3	Forward	AGGTTTGCTCAGGGGTTCATA	NM-004369.4
	Reverse	AGCCGCACCATTTTTGACAT	
LAMA1	Forward	CTTCCTGAAAGGCGGCTACA	NM_005559.4
	Reverse	TGTGCTTCCTCACGATCACC	
LAMB1	Forward	TGTGGTGGTCTGGTTACTGTT	NM_002291.3
	Reverse	TCAGTTTTGCTTCAGAGACCATCT	
LAMC1	Forward	AGCGCTGTCCAAAAGAATGC	NM_002293.4
	Reverse	CCTGGTCAGCGTCATCTTGT	
FN1	Forward	CTATCACCTGTACCCACACGG	NM_212482.4
	Reverse	CCAGGAACCCTGAACTGTAAGG	
NID1	Forward	CGGATTCCGTGAGAGGGAAC	NM_002508.3
	Reverse	AGGTCAGTCCATTGGGCAAG	
ELN	Forward	CATTTCTCCCCGAGATGGCG	NM_000501.4
	Reverse	CGAGACCAGCCCCTGGATAA	
